# Evaluation of Vitamin D_3_ and D_2_ Stability in Fortified Flat Bread Samples During Dough Fermentation, Baking and Storage

**DOI:** 10.15171/apb.2017.038

**Published:** 2017-06-30

**Authors:** Mehrnaz Tabibian, Mohammadali Torbati, Mohammad Reza Afshar Mogaddam, Maryam Mirlohi, Malihe Sadeghi, Javad Mohtadinia

**Affiliations:** ^1^Department of Food Science and Technology, Faculty of Nutrition and Food Sciences, Tabriz University of Medical Sciences, Tabriz, Iran.; ^2^Pharmaceutical Analysis Research Center, Tabriz University of Medical Sciences, Tabriz, Iran.; ^3^Department of Food Science and Technology, Faculty of Nutrition and Food Sciences, Isfahan University of Medical Science, Isfahan, Iran.; ^4^Department Food Hygiene and Safety, Faculty of Nutrition and Food Sciences, Isfahan University of Medical Sciences, Isfahan, Iran.

**Keywords:** Bread sample, Vitamin D_3_, Vitamin D_2_, High performance liquid chromatography

## Abstract

***Purpose:*** Vitamin D, a fat-soluble secosteroid, has a significant role in bone metabolism and helps calcium absorption in the body. Since vitamin D concentration is altered in fortified foods and dietary supplements, the actual amount of vitamin D may differ from the label value.

***Methods:*** In this study, the concentrations of vitamin D_2_ and D_3_ of fortified bread sample were analytically determined. For this purpose, dough or homogenized bread sample was saponified using potassium hydroxide solution (30%, w/v) at 80°C, and the saponified analytes were extracted into *n*-heptane followed by liquid-liquid extraction. Then *n*-heptane fraction was evaporated to dryness and the sample was reconstituted in methanol. The effect of different parameters was evaluated by one variable at one-time strategy.

***Results:*** The analytes concentrations were evaluated in dough fermentation, baking and storage steps. The effect of temperature in dough fermentation and baking was evaluated at the range of 5-30 and 200-250°C, respectively. Also, the fermentation time was studied in the range of 0-120 min. The analytes concentrations were followed for 1 to 5 days after baking. The results indicated that dough fermentation temperature has no significant effect on the concentration of the analytes. On the other hand, when the dough fermentation time and baking temperature are increased, the analytes concentrations are decreased. Also, the storage duration of the spiked bread samples decreased the analytes concentrations after one day.

***Conclusion:*** Based on the obtained results, baking the dough at high temperatures lead to decrease in vitamin levels.

## Introduction

Vitamins are essential for the health of humans and animals and cannot be synthesized by these vertebrates. Therefore, they must be obtained from the diet. Generally, vitamins are classified into categories named fat-soluble or water-soluble according to their solubilities in solvents.^1^ Fat-soluble vitamins are composed of four vitamins namely A, E, D, and K.^2^ Among these compounds vitamin D has a key role in human health. Vitamin D deficiency is known as a major hidden health problem worldwide. During recent years there has been increasing evidence indicates that vitamin D may contribute to the pathogenesis of multiple sclerosis and influence the disease course and activity.^3^ On the other hand, excessive vitamin D intake is associated with the risk of hypercalcaemia or hypercalciuria and kidney problems.^4,5^ Human obtains his vitamin D needs in two forms including vitamins D_3_ (cholecalciferol) and D_2_ (ergocalciferol) from two sources. Cholecalciferol is biosynthesized in the skin during exposure to UV light, whereas ergocalciferol is absorbed from the diet.^6^ Although animal originated foods are traditionally supplemented by excess level of vitamin D_3_, fortifying different foods has recently been considered. One of the most important foods is bread.^7^ Bread is a main food prepared from the dough of flour and water, typically by baking. In some bread, non-cereal materials including nuts, fats and vitamins are added to improve the nutritional value due to high consumption of it in different meals.^8-10^ After fortification, the most crucial parameter is the stability of the added materials in different procedures. Vitamin D stability in fortification regimes has been investigated in milk, yogurt and cheese.^11-13^ The obtained results indicate that vitamin D stability in pure form decreases by heating to 150 °C in the presence of air. On the other hand, in acidic media vitamin D might be isomerised to isotachysterol. Similar to these products vitamin D can be altered in bread samples during dough fermentation, baking, and storage which should be considered.^14-16^

The main goal of this study is the investigation of vitamin D contents in Iranian flat bread (Taftoon) prepared during fermentation, baking and storage. For this purpose, bread samples were spiked with vitamin D_3_ and D_2_ and then they were determined using high performance liquid chromatography-ultraviolet detector (HPLC-UV). The samples were extracted with the protocol provided by the Institute of Standards and Industrial Research of Iran entitled as “Foodstuffs-determination of vitamin D by high performance liquid chromatography; measurement of vitamin D_3_ and D_2_ (document No. 13579)”. The influence of different parameters was optimized. Finally, the recommended method was applied to the determination of the selected analytes from the bread samples.

## Materials and Methods

### Chemicals and solutions

The selected vitamins including D_3_ and D_2_ powders with the purity higher than 99% were obtained from DSM Company (Kaiseraugst, Switzerland). High purities of ascorbic acid, hydrochloric acid, sodium chloride, potassium hydroxide, ethanol, and* n*-heptane were purchased from Merck (Darmstadt, Germany). HPLC-grade methanol and water were purchased from Merck. A 50 mg L^-1^ of standard stock solution of the analytes was prepared in ACN and it was stored in a refrigerator at 4 °C. A standard solution of analytes (50 mg L^−1^ of each analyte) in mobile phase was injected into the analysis system (three times a day) for quality control and the obtained analytical signals (peak areas) were used for the calculation of extraction recoveries (ERs).

### Instrumentation 

A Jasco PU-2080 plus HPLC (Tokyo, Japan) was equipped with a senary pump, degasser, automatic injector (AS-2055 Plus), and UV-Vis detector (UV-2075 plus). A Macherey-Nagel analytical C18 column (150 × 4.6 mm) (Germany) packed with 5 µm particles was used for the separation. The mobile phase was methanol: water (95: 5, v/v) delivered at a flow rate of 1 mL min^-1^. The analytes were monitored at 265 nm.‏ Data acquisition and processing were done using ChemStation software.

### Preparation of bread sample

At first, 1000 g of wheat flour with 88% extraction rate (the amount of flour which is extracted from a given amount of wheat) was provided from local supermarkets (Tabriz, Iran), was mixed with 20 g dry yeast, and 10 g sodium chloride. Then 850 mL HPLC-grade water was added to the mixture. After mixing manually, the mixture was spiked 100 ng g^-1^ (each analyte) of vitamin D_3_ and D_2_. The obtained dough was left to rise for 60 min at room temperature. Before baking, 250 g of the dough was taken for analysis. In the final stage, the fermented dough was flattened out and cooked at 220 °C for 15 min.

### Sample preparation

Sample preparation was performed according to Institute of Standards and Industrial Research of Iran protocol (document No. 13579). For this purpose, 20 g of dough or homogenized bread sample was transferred into a flask and 100 mL ethanol, 2 g ascorbic acid and 50 mL potassium hydroxide solution (30%, *w/v*) were added to the flask. The mixture was adjusted at 80°C and was refluxed under N_2_ stream to saponification of the analytes. After cooling the mixture (to room temperature) with cool water, the samples were added to separatory funnel and extracted into *n*-heptane (50 mL). To remove water residue from *n*-heptane, one spatula of sodium sulfate was added to the mixture and then it was evaporated using rotary vacuum system. Thus, the residue was dissolved in methanol, and to remove interferences, the mixture was passed from SPE silica gel columns. The eluant from column evaporated, and the residue was dissolved in methanol and injected into the separation system for the analysis.

## Results and Discussion

### Optimization of mobile phase composition and flow rate

The comparison of vitamin D_3_ and D_2_ separation under different mobile phase compositions was performed with the calculation of resolution factor (R_s_) of two peaks. R_s_ was calculated based on the following equation: R_s_ = 1.18 (t_R2_ − t_R1_)/(W_1_ +W_2_) where t_R2_ and t_R1_ are the retention times of two peaks, and W_1_ and W_2_ are the peak widths at 50% of their maximum heights.^17^ Baseline separation was achieved when R_s_ = 1.5. For this purpose, optimization of mobile phase composition was performed by direct injection of the target analytes at a concentration of 25 mg L^-1^ (each analyte). For this purpose, methanol and water compositions were altered at the ratios of 100:0, 95:5, 90:10, and 85:15, v/v. According to the results, better resolution factor and peaks shape were obtained at the ratio of 95: 5, v/v, methanol: water. Therefore 95: 5, v/v, methanol: water was selected as suitable mobile phase for further experiments. Flow rate of the selected mobile phase was studied at the range of 0.7-1.3 mL min^-1^. The data showed that by increasing the flow rate up to 1 mL min^-1^, Rs increased and then decreased. As a result, the flow rate was adjusted at 1 mL min^-1^.

### Analytical features of the method

Under optimal conditions, the analytical characteristics of the developed method were evaluated. These parameters include LODs, the correlation coefficients of determination (r), limits of quantification (LOQs), relative standard deviations (RSDs), linear ranges (LRs) of calibration graphs and ERs were tabulated in Table 1. The wide LRs were obtained with correlation coefficients at the range of 0.996 to 0.998. The LODs, calculated on the base of S/N of 3, were 22.3 and 19.5 ng mL^-1^ for vitamin D_2_ and D_3_, respectively. The LOQs (S/N=10) were 78.2 and 67.4 ng mL^-1^ for vitamin D_2_ and D_3_, respectively. RSDs for spiked samples at 100 ng g^-1^ of each analyte were in the ranges of 4.3-6.7% and 5.9-7.9% for intra-day (n=6) and inter-day (n=4) precisions, respectively. The accuracy of the method was determined by standard addition method using at 100 and 300 ng mL^-1^ levels (each analyte, n=5), and the obtained deviations were less than 8% for all analytes. These values show that the method is responsive and suitable for the determination of the selected analytes in bread samples.

**Table 1:** Quantitative features of the developed method for the selected vitamins.

**Vitamins**	**Equation**	**LOD** ^a)^	**LOQ** ^b)^	**LR** ^c)^	**r** ^d)^	**RSD %** ^e)^		**ER%** ^f)^
						**Intra-day**	**Inter-day**	
D_2_	Y=890x+98	22.3	78.2	78.2– 25000	0.996	4.3	5.9	75 ± 3
D_3_	Y=1980x+6	19.5	67.4	67.4 – 25000	0.998	6.7	7.9	82 ± 4

a) Limit of detection (S/N=3**) (**ng mL^-1^) b) Limit of quantification (S/N=10) (ng mL^-1^) c) Linear range (ng mL^-1^) (n=7) d) Correlation coefficient e) Relative standard deviation (n=6, C =100 ng mL^-1^) for intra-day and (n= 4, C=100 ng mL^-1^) for inter-day f) Extraction recovery

### Optimization of dough fermentation time on vitamin D_3_ and D_2_ concentration 

**Figure 1:**
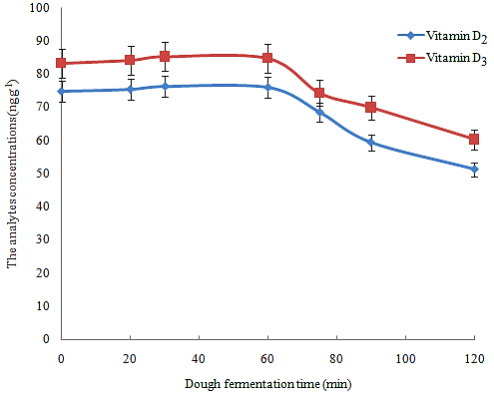
Optimization of dough fermentation time on the concentrations of the analytes. Extraction condition: dough sample, 20 g; dough fermentation time, 0-120 min; ascorbic acid 2 g; potassium hydroxide solution volume, 50 mL; potassium hydroxide solution concentration, 30%, w/v; reflux temperature, 80°C ; extraction solvent, *n*-heptane; extraction solvent volume, 50 mL. The error bars indicate the minimum and maximum of three determinations.

One of the most important steps of bread making is dough fermentation. Fermentation occurs when yeast and bacteria inside the dough convert carbohydrates to carbon dioxide causing gas bubbles to form, which has a leavening effect on dough. When bacteria and yeast of the dough convert carbohydrates to carbon dioxide, fermentation happens and gas bubbles are formed in the dough. Therefore, the dough appears larger and larger. Due to the sensitivity of the selected analytes, this step may affect the spiked vitamin concentrations. To study the effect of dough fermentation time on the selected analytes concentrations, the prepared dough was left at 0, 20, 30, 60, 75, 90, and 120 min. The obtained results in Figure 1 demonstrated that by increasing the fermentation time up to 60 min, the analytical signals are constant and then decreased, which can be attributed to the oxidation of vitamins. Therefore, 60 min was suitable time for dough fermentation.

### Optimization of dough fermentation temperature on vitamin D_3_ and D_2_ concentration

Dough fermentation is affected by temperature. On the other hand, spiked vitamin concentrations may alter at different temperatures. To evaluate the dough fermentation temperature on the spiked vitamin concentrations, the prepared dough was left at 5, 10, 20, 23 (room temperature), and 30 °C at 60 min. After the preparation method on the dough samples was performed, the obtained results indicated that temperature change has no significant effect on the performance of the method. Subsequently, further experiments were performed at room temperature.

### Optimization of baking temperature on vitamin D_3_ and D_2_ concentration

**Figure 2:**
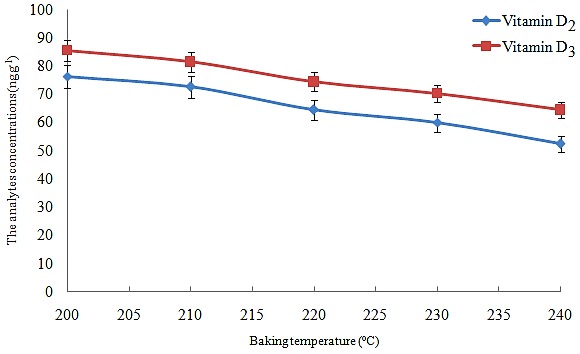
Effect of baking temperature on the concentrations of the analytes. Extraction condition: are the same as Figure 1, except dough fermentation time was adjusted at 60 min. The error bars indicate the minimum and maximum of three determinations.

Another important parameter which can affect the selected analytes concentration is baking temperature. The prepared dough was baked at the range of 200-250°C (at 10°C intervals) for 15 min. After performing the method, the obtained results in Figure 2 showed that by increasing the baking temperature, analytes concentrations were decreased (nearly 25%), which was attributed to the decomposition of vitamins in higher temperatures. It is noted that at temperatures lower than 200°C, the prepared dough was not cooked completely and the method became useless.

### Evaluation of storage duration of bread on vitamin D_3_ and D_2_ concentration

After the baking of bread, storage duration of the bread can affect its shelf life and consequently the concentration of the additives. During storage, its crumb firms due to a combination of different events and to a degree determined by the strength of the different networks in the system. Fresh bread at ambient temperature contains a semi-crystalline amylose network and a thermoset gluten network. However, during the storage of bread these, networks are changed and the quality of the bread is decreased. On the other hand, the fortified components like vitamins may alter. To evaluate vitamin D_3_ and D_2_ concentrations the method was performed on bread samples after one, three, and five days of storage in room temperature at three concentrations including 100, 300, and 500 ng mL^-1^ (each analyte). It was noted that the samples were covered with freezer bags under ordinary light. The obtained recoveries against fresh bread at the same concentrations are demonstrated in Table 2. The data indicated that the recoveries were decreased by increasing storage duration from 1 to 5 days. It is remarkable that vitamin D_3_ and D_2_ are sensitive to the oxidation and degradation under air stream or UV radiation. Storage temperature may affect the spiked vitamin’ concentrations in the samples.

**Table 2:** Mean relative recoveries of the selected analytes after storage duration

**Vitamin**	**Mean relative recoveries ± standard deviation (n=3)**		
	**1 day**	**3 days**	**5 days**
	All samples were spiked with each analyte at a concentration of 100 ng mL^-1^.		
D_2_	98 ± 3	86 ± 3	79 ± 3
D_3_	99 ± 2	89 ± 5	72 ± 4
	All samples were spiked with each analyte at a concentration of 300 ng mL^-1^.		
D_2_	99 ± 5	92 ± 3	81 ± 3
D_3_	100 ± 4	89 ± 5	80 ± 3
	All samples were spiked with each analyte at a concentration of 500 ng mL^-1^.		
D_2_	101 ± 5	91 ± 6	85 ± 3
D_3_	97 ± 6	90 ± 3	84 ± 4

### Applicability of the method

**Figure 3:**
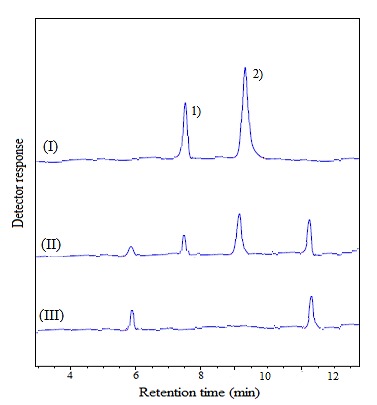
HPLC-UV chromatograms of (I) direct injection of standard solution of the selected analytes in methanol at concentration of 25 mg L^-1^(each analyte), (II) spiked bread sample at concentration of 100 ng mL^-1^ (each analyte) after performing the developed method, and un-spiked sample after performing the developed method. Detection wavelength, 265 nm. For chromatographic conditions see experimental section. Peaks identification: 1) Vitamin D_2_, and 2) Vitamin D_3_.

In order to demonstrate the feasibility of the developed method, it was used to determine the target analytes in fortified bread samples. Figure 3 depicts typical chromatograms of the selected samples after performing the proposed method. To evaluate the matrix effect on real samples, standard addition method was used. The samples were fortified with the target analytes at two concentrations (100 and 200 ng mL^−1^ of each analyte) and the developed method was performed on them (three times for each concentration). The found values are mentioned in Table 3. The obtained results showed that the matrices of samples have a relatively low effect on the performance of the developed method. On the other hand, to evaluate the loss of the analytes during the process out of the bread matrix a blank dough sample was spiked with analytes at the concentration of 100 ng g^-1^ and it was analyzed without fermentation. The found values for the analytes showed that there was no significant difference between the added and found concentrations. Selectivity of the proposed method was studied by the analysis of three bread samples obtained from different sources. As it can be concluded from Figure 3, there was no interfering peak in retention times of the analytes, and the developed method was selective for the analytes. On the other hand, these samples were analyzed at four wavelengths including 220, 254, 260, and 270 nm and the obtained results showed that there was no peak in these wavelengths and the method was specific for the analytes.

## Conclusion

In the present work, the stability of vitamin D_3_ and D_2_ has been studied in bread samples in different conditions. The obtained results showed that the added vitamins’ level was decreased at the fermentation time higher than 60 min. However, the added vitamins are stable at different temperatures in this step. Baking the dough at high temperatures led to decrease in vitamin levels. Also, vitamin D_3_ and D_2_ levels in stale bread were less than fresh bread.

**Table 3:** Results of assays to check the samples matrices effect for the selected analytes

**Vitamin**	**Added (ng mL^-1^)**	**Found (ng mL^-1^)**			
		**Sample 1**	**Sample 2**	**Sample 3**	**Sample 4**
D_2_	100	98.9 ± 0.4	99.0 ± 0.2	9‏.6 ± 0.3	9‏.4 ± 2.4
	200	195.5 ± 5.5	192.3 ± 4.3	197.5 ± 6.3	195.5 ± 5.5
D_3_	100	94.1 ± 0.8	95.1 ± 0.7	9.2 ± 0.2	98.9 ± 3.6
	200	199.2 ± 1.1	196.6 ± 1.4	198.1 ± 0.4	195.5 ± 4.3

## Acknowledgments

Authors present their appreciation to the Tabriz University of Medical Sciences for their financial support of this project (No. 40/A/93/8/15).

## Ethical Issues

Not applicable.

## Conflict of Interest

The authors declare no conflict of interests.
